# Effects of taking a nap or break immediately after night shift on nurses’ fatigue recovery and sleep episodes: a quasi-experimental study

**DOI:** 10.1186/s40101-025-00399-2

**Published:** 2025-07-15

**Authors:** Issei Konya, Inaho Shishido, Kazuhiro Watanabe, Masayuki Ikebuchi, Toshiyasu Tanaka, Hisao Kataoka, Rika Yano

**Affiliations:** 1 Division of Nursing, Hokkaido University Hospital, Kita 14, Nishi 5, Kita-ku, Sapporo, 060-8648 Japan; 2 Faculty of Health Sciences, Hokkaido University, Kita 12, Nishi 5, Kita-ku, Sapporo, 060-0812 Japan; 3 Graduate School of Health Sciences, Hokkaido University, Kita 12, Nishi 5, Kita-ku, Sapporo, 060-0812 Japan; 4 Lighting Development Center, Electric Works Company, Panasonic Corporation, 1048 Kadoma, Kadoma City, Osaka, 571-8686 Japan

**Keywords:** Fatigue, Nurses, Night shift, Shift work, Sleep, Recovery, Quasi-experimental studies

## Abstract

**Background:**

Excessive fatigue, sleep deprivation, and poor intershift recovery after night shifts are associated with an increased risk of traffic accidents and maladaptive chronic fatigue. However, little is known about whether taking a nap or break immediately after a night shift improves fatigue recovery and sleep among nurses. This study aimed to examine the effects of taking a 30-min nap or break immediately after a 16-h night shift on fatigue recovery and sleep episodes among nurses.

**Methods:**

A quasi-experimental crossover study was conducted with 62 nurses who worked 16-h night shifts. Nurses were randomly assigned to two condition sequences (AB or BA): (A) intervention (the nurses took a 30-min nap or break in a nap environment control system after a night shift) and (B) control (the nurses went home as usual after a night shift). Fatigue was measured immediately after the night shift, after taking a nap or break, and after getting up from nighttime sleep on the day after the night shift. Sleep episodes were assessed using a wearable device. Outcomes were compared between the two conditions.

**Results:**

In the intervention condition, fatigue immediately after the night shift was significantly reduced by taking a nap or break, with a large effect size. Recovery from “local pain or dullness,” one of the symptoms of work-related fatigue, was significantly higher in the intervention than control condition. Compared to the control condition, the intervention condition significantly delayed the timing of the first sleep episode, whereas no significant differences were observed in the main sleep parameters.

**Conclusions:**

Taking a nap or break immediately after the night shift could be a countermeasure to address fatigue/sleep-related problems among nurses. For nursing managers, encouraging nurses to take a nap or break immediately after the night shift could be a viable option in comprehensive fatigue risk management. Therefore, appropriate napping environments should be installed in clinical settings.

Trial registration: UMIN000038444 (date of registration: 30 Oct 2019)

**Supplementary Information:**

The online version contains supplementary material available at 10.1186/s40101-025-00399-2.

## Background

Hospital nurses engaged in shift work are indispensable in ensuring continuous 24-h care for patients [1]. However, shift work, especially night shifts, disrupts nurses’ circadian rhythms, leading to sleep deprivation and excessive fatigue [2]. According to the two-process model of sleep regulation [3], circadian misalignment can further contribute to sleep deprivation, as there is a reduced homeostatic drive to sleep during the daytime following a night shift [4]. Moreover, other daily obligations such as family responsibilities can further shorten sleep time after a night shift [5]. Lack of sleep and poor sleep quality associated with night shifts cause excessive fatigue and various health problems in nurses [6, 7]. This vicious cycle poses a serious threat to the quality of nursing care and patient safety [8, 9].

As a countermeasure against the adverse outcomes of night work, napping on night shifts may mitigate nurses' sleep deprivation and promote the regulation of circadian rhythms [10]. Appropriate night-shift napping prevents daytime sleepiness [5, 10], reduces fatigue during night shifts [11], and promotes post-work recovery from fatigue [12]. However, night-shift napping alone cannot solve shift nurses' fatigue and sleep problems, and further research that incorporates a more comprehensive fatigue risk management system is needed [2]. Since appropriate recovery from fatigue after a night shift prevents maladaptive chronic fatigue [13], it is important to examine the optimal approaches to sleeping after a night shift. Napping after a night shift—the subject of this study—is potentially an important part of an overall fatigue recovery framework.

Sleep deprivation immediately following the night shift adversely affects nurses’ cognitive functions, including visuospatial and verbal memory [4]. Therefore, night shifts are associated with an increased risk of dozing off while driving, driving off the road, and being involved in a motor vehicle crash [14, 15]. Scott et al. [16] reported that 596 out of 895 nurses had experienced at least one drowsy driving episode in the past 4 weeks. Additionally, longer working hours (≥ 12-h) significantly increase the risk of drowsy driving and motor vehicle crashes [16]. Thus, previous studies have suggested that nurses should pay special attention to driving home and take a short nap at the end of the night shift before returning home [15, 16].

The quality of the first and main sleep episodes at home was associated with fatigue recovery from the night shift to the next day, regardless of age [17]. This is crucial, given that poor intershift recovery can transform acute to chronic fatigue. Maladaptive chronic fatigue can lead to depression and reduced concentration and motivation [18], as well as lower quality of care and higher turnover [19, 20]. If napping immediately after the night shift can reduce acute fatigue before returning home, fatigue may remain low, and the possibility of pursuing leisure activities in non-working hours may increase. Therefore, we hypothesized that napping immediately after the night shift would facilitate recovery from fatigue after returning home, in part by facilitating leisure activities during non-working hours that can aid the recovery process [21, 22].

During night-shift napping, environmental factors, such as napping space, napping duration, noise, and light levels, affect the quality of the nap [2, 10, 23, 24]. Since napping environments in clinical settings vary [11], van Woerkom [25] examined the effect of napping on fatigue during night shifts using a nap facility that controls the napping environment. Similarly, we used a nap environment control system in this study.

This study aimed to examine the effects of taking a nap or break immediately after a long night shift on fatigue recovery and sleep episodes among nurses. The findings may provide a novel countermeasure for sleep deprivation and fatigue immediately after long night shifts, mitigating the various risks associated with these conditions. The main hypotheses are as follows:

1. Nurses are less fatigued after taking a nap or break than immediately after their night shift.
2. Nurses who take a nap or break after the night shift have higher fatigue recovery (from immediately after the night shift to getting up from nighttime sleep on the day after the night shift) than nurses who go home as usual.
3. Nurses who take a nap or break have better sleep parameters during sleep episodes at home than those who go home as usual.

## Methods

### Study design and setting

A quasi-experimental crossover study was conducted from December 2019 to March 2020 in a general hospital (six wards, over 200 beds) in northern Japan. This hospital operates on a two-shift system, including a 16-h night shift (16:30–9:00). In principle, each nurse was allocated a single scheduled nap break lasting at least 2-h per night shift. However, the start time, maximum duration, and order of nap breaks were adjusted as necessary among nurses according to work demands.

The nurses experienced the following two night-shift conditions: (A) intervention condition (after working the 16-h night shift, the nurses took a 30-min nap or break in a nap environment control system before going home) and (B) control condition (after working the 16-h night shift, the nurses went home as usual). We randomly assigned nurses to one of two sequences (AB or BA). We avoided assigning conditions to shift schedules with two consecutive night shifts or vacations, because the number of rest days after the night shift would have been a confounding factor. Moreover, the survey was conducted on weekdays to ensure there were no differences in work demands between the conditions. However, the nature of the intervention made it impossible to conduct double blinding.

The study was prospectively registered on the University Hospital Medical Information Network (registration no. UMIN000038444) and was performed following the Transparent Reporting of Evaluations with Non-Randomized Designs (TREND) statement (Additional file 1) [26].

### Participants

**Fig. 1:**
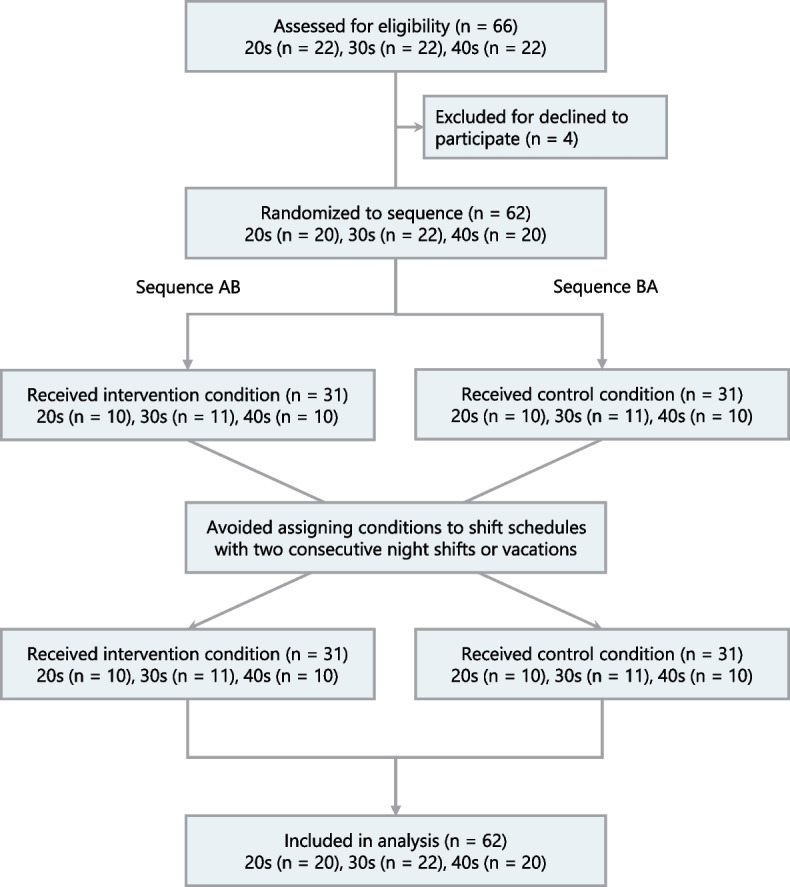
Study flow diagram

This study included healthy registered nurses aged from 20 to 49 years who worked 16-h night shifts. The exclusion criteria were as follows: (1) nurses with less than 1 year of experience, (2) receiving any form of medical treatment, (3) regularly used sleeping pills, (4) pregnant, and (5) a history of claustrophobia or epileptic seizures. We used stratified sampling by age (20s, 30s, and 40s), as age affects nurses’ fatigue and sleep [21]. Based on the eligibility criteria, we recruited 66 nurses (22 in each age group), which was the maximum number that could be included in the target facility. The study flow diagram, including the random assignment process in each age category, is illustrated in Fig. 1. Sample size calculations in analysis of covariance (ANCOVA) were performed using G Power software ver. 3.19 [27], assuming a medium effect size (*f* = 0.25) according to previous studies [25, 28], *α* = 0.05, and 1-β = 0.80. The minimum sample size for each condition was 64; hence, our study sample was within the acceptable range.

### Intervention

In the control condition, nurses returned home immediately after completing their 16-h night shift, and followed their usual routine without any intervention. The intervention condition involved using a nap environment control system immediately after the night shift to take a 30-min nap or break. We provided the “nap environment control system (Panasonic’s prototype, system that provides a space suitable for napping: 1680 mm × 2300 mm × 1735 mm).” Previous studies have suggested that the optimal length for a nap is less than 30-min, as sleep inertia or a brief period of impaired alertness is more likely to occur after substantial sleep (e.g., more than 30-min) [29, 30]. Therefore, this system was selected so that the same quality of nap or break interventions could be implemented. The system uses technology to control environmental light, sound, scent, and airflow to promote sleep onset and comfortable awakening after 30-min. The 30-min period of operation consists of sleep onset, nap, and awakening phases. The interior has a reclining chair that can be adjusted to a comfortable position by the user. The researchers and research assistants performed all operations related to the use of the system. To counter the primacy effect, all participants had experienced taking a nap or break in the system once before starting the study.

### Outcomes

**Fig. 2:**
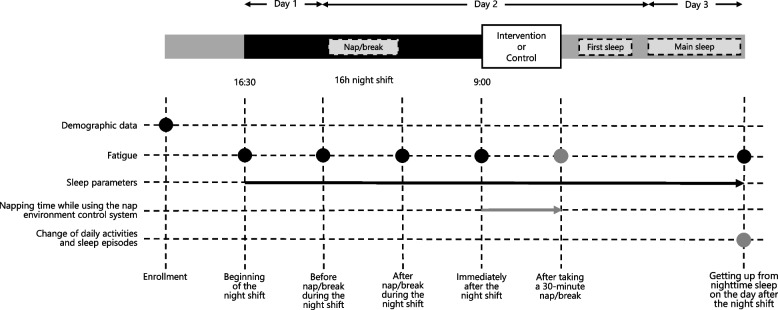
Study protocol Notes: black circles, measurements; black arrow, continuous measurement; gray circles, measurements in the only intervention condition; gray arrow, continuous measurement in the only intervention condition; dotted areas, individuality among nurses

The study protocol and data collection schedule are illustrated in Fig. 2.

#### Fatigue

Work-related fatigue was measured by the “Jikaku-sho shirabe” [31]. This scale was developed by the Working Group for Occupational Fatigue, part of the Japan Society for Occupational Health, and is widely used to measure acute fatigue in shift-work nurses [11, 17, 32]. Participants answered 25-items across 5-factors (feeling of drowsiness, instability, uneasiness, local pain or dullness, and eyestrain) on 5-point Likert scales from 1, “completely disagree,” to 5, “strongly agree.” Higher scores indicate a higher level of acute fatigue; both total scores and factor scores can be used in analyses. Participants responded to the scale at the beginning of the night shift (Day 1) and before and after the scheduled nap or break period on the night shift (Day 2), during which some nurses took a self-initiated nap while others chose to remain awake. Additionally, as key measurement points, participants responded immediately after the night shift (Day 2), after taking a 30-min nap or break following the night shift (intervention condition only; Day 2), and on getting up from nighttime sleep on the day after the night shift (Day 3).

#### Sleep parameters

Sleep parameters in night-shift napping, first sleep episodes, and main sleep episodes were calculated using objective and subjective data. The first and main sleep episodes were defined as “the first sleep episode after the night shift, excluding the main sleep episode” and as “the longest sleep episode from immediately after the night shift to getting up from nighttime sleep on the day after the night shift,” respectively [17].

MTN-220 (ACOS CO., LTD., Iida, Japan) was used for objective sleep assessment [33]. This wearable device (diameter, 27.0 mm; thickness, 9.1 mm; weight, 9.0 g) records activity and posture using a built-in 3-axis accelerometer. Nurses were asked to wear the device on the front of their trunk (trousers) for three consecutive days: from the beginning of the night shift (Day 1) to getting up from nighttime sleep on the day following the night shift (Day 3). The recorded data were analyzed using SleepSign Act ver. 2.0 (SSA) software (KISSEI COMTEC CO., LTD., Matsumoto, Japan).

A sleep diary was used to assess subjective sleep. The participants were asked to record sleep data that the researcher could not observe directly, such as nap or break times during the night shift, sleep time after returning home, and times the wearable device was removed.

The definitions of each sleep parameter are as follows.

1. Time in bed (TIB; min): total time a person spent lying in bed.
2. Sleep latency (SL; min): the time from lying to falling asleep.
3. Total sleep time (TST; min): the sum of periods a person was asleep between the start and end of TIB.
4. Sleep efficiency (SE; %): the ratio of TST to TIB.
5. Wake after sleep onset (WASO; min): the sum of time spent awake during a sleep.
6. Bed-out time (BOT; min): the time between waking up and getting up.

By setting “start of TIB” and “end of TIB” using objective and subjective sleep data [17], other sleep parameters were automatically calculated according to the SSA algorithm [34].

#### Napping time while using the nap environment control system

An Early Sense System (EarlySense, Ltd., Israel), an already validated contact-free sleep monitoring device [35], was inserted under the reclining chair in the nap environment control system to assess the participants’ total napping time (min) while using the system.

#### Change of daily activities and sleep episodes after taking a nap or break

Under the intervention condition, participants were asked whether there were “changes of daily life, activities, and main sleep episodes” after the night shift. Those who responded “yes” were asked to freely describe how taking a nap or break changed their daily life, activities, and main sleep episodes.

#### Demographic data

At the beginning of the study, participants completed questionnaires regarding age, years working as a nurse, body mass index, sex, and family and domestic responsibilities (marital status or child rearing).

### Statistical analysis

Continuous variables were presented as medians and interquartile ranges (IQR), while categorical variables were presented as frequencies and percentages. The normality of each variable was assessed using the Shapiro–Wilk test. Statistical analyses were performed using JMP^®^16 Pro (SAS Institute Inc., Cary, NC, USA), with a significance level of 5%.

A mixed model was employed as a preliminary analysis to confirm that the time course of fatigue during the night shift was similar between the two conditions (Additional file 2). In this model, the dependent variables were fatigue scores, while the fixed effects were condition (intervention and control), time (pre-night shift, pre-nap/break, post-nap/break, and post-night shift), and their interactions. Random effects were nurses nested by ward (nurses [wards]). Subsequently, the analyses were conducted in four stages corresponding to the hypotheses.

To test Hypothesis 1, the Wilcoxon signed-rank test and Bonferroni correction were used to compare each fatigue score within the time points in the intervention condition. Effect sizes were calculated as *r* =|z|/√n using the z statistic; effect sizes of 0.10, 0.30, and 0.50 were considered small, medium, and large, respectively [36].

To test Hypothesis 2, a mixed model was employed to compare the degree of fatigue recovery between the two conditions. Changes in each fatigue score (immediately after the night shift—getting up from nighttime sleep on the day after the night shift) were set as the dependent variable, while nurses nested by ward (nurses [wards]) were set as the random effect. Fixed effects were the conditions, with each fatigue score immediately after the night shift (baseline value) included as a covariate. Partial *η*^*2*^ was calculated for the effect size; effect sizes of 0.01, 0.06, and 0.14 were considered small, medium, and large, respectively [37]. In addition to stratified sampling to adjust for the effect of age between the two conditions, we conducted an exploratory analysis to evaluate the effect of the interactions between condition and age on fatigue recovery.

As a subgroup analysis for Hypothesis 2, we explored possible heterogeneity related to family responsibilities and night-shift napping. A previous study reported that night-shift napping promoted fatigue recovery [12]. A recent scoping review of factors impeding fatigue recovery at home among shift nurses identified family and domestic responsibilities as potential factors [21]. Thus, the interactions of the three factors of condition, family role, and night-shift napping were added to the mixed model.

To test Hypothesis 3, the Wilcoxon signed-rank test was used to compare the first and main sleep episodes between the two conditions. The first and second authors independently categorized the free-text descriptions from the sleep diaries regarding how taking a nap or break after the night shift influenced daily life, activities, and sleep episodes. The contents were then consolidated, and discrepancies were resolved through discussion with all co-authors to reach a consensus on the final categories. The percentage of responses in each category was then calculated and summarized.

## Results

### Participants’ characteristics and napping time while using the nap environment control system

Of the 66 nurses recruited, 4 withdrew, leaving a total of 62 nurses for analysis (Fig. 1). Table 1 summarizes participant characteristics; 20 nurses (32.3%) were in their 20s, 22 nurses (35.5%) were in their 30s, and 20 nurses (32.3%) were in their 40s. Eighteen nurses (29.0%) had family responsibilities. The napping rate during night shifts (approximately 85%) was analogous among the conditions.

**Table 1:** Participants’ characteristics

	Control (*n* = 62)	Intervention (*n* = 62)
Age (years)	34.0 (28.0–42.0)	
Years as a nurse (years)	9.8 (4.9–16.9)	
Body mass index (kg/m^2^)	21.1 (19.3–22.2)	
Sex (female)	60 (96.8%)	
Family and domestic responsibilities at home (yes)	18 (29.0%)	
Married (yes)	16 (25.8%)	
Child-rearing (yes)	13 (21.0%)	
Cumulative steps during night shift (steps)	9848.5 (7577.0–11,826.3)	9540.0 (8072.5–11,636.5)
Resting time during night shift (min)	120.0 (120.0–150.0)	120.0 (115.0–155.0)
Napping during night shift (yes)	52 (83.9%)	55 (88.7%)
Start of TIB (hh:mm)	26:11 (25:03–27:22)	26:28 (24:55–27:17)
TIB (min)	90.0 (68.0–112.0)	113.0 (91.5–144.0)
SL (min)	6.0 (4.0–14.0)	18.0 (6.0–28.0)
TST (min)	66.0 (52.0–89.0)	80.0 (59.5–104.0)
SE (%)	80.5 (70.7–88.6)	68.8 (57.4–80.6)
WASO (min)	4.0 (0.0–10.0)	6.0 (0.0–16.0)
BOT (min)	4.0 (2.0–6.0)	4.0 (4.0–6.0)
End of TIB (hh:mm)	27:55 (26:36–28:56)	28:50 (26:43–29:22)

Notes: Continuous variables were presented as medians (interquartile ranges), whereas categorical variables were described as *N* (%)

Abbreviations: *TIB*, the total time a person spent lying in bed; *SL*, the time from lying to falling asleep; *TST*, the sum of periods a person was asleep between the start and end of TIB; *SE*, the ratio of TST to TIB; *WASO*, the sum of time spent awake during a sleep; *BOT*, the time between waking up and getting up

**Fig. 3:**
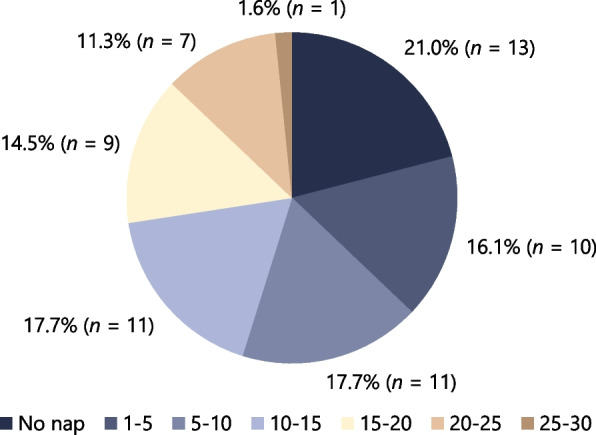
Percentage of total napping time (min) while using nap environment control system

Figure 3 shows the proportion of total nap time under the intervention condition; 21.0% (*n* = 13) simply took a break rather than napping while using the nap environment control system. We explored potential differences in participant characteristics, sleep, and fatigue recovery between those who napped and those who only took a break (Additional file 3). However, owing to the small sample size and potential imbalance between subgroups, we did not conduct separate analyses and draw definitive conclusions regarding the differential effects of naps versus breaks.

### Fatigue recovery

In multiple comparisons within the intervention, the total fatigue score after taking a nap or break was significantly lower than that immediately after the night shift, with a large effect size (*r* = − 0.74, *P* < .001; Table 2). Similar trends were observed for each fatigue sub-factor. Thus, Hypothesis 1 was confirmed.

**Table 2:** Change in fatigue after taking a nap or break (intervention condition)

Variables	T1: Immediately after the night shift	T2: After taking a 30-min nap or break	T3: Getting up from nighttime sleep on the day after the night shift	Comparisons	*r*	*P*
Total score	55.0 (42.0–80.0)	43.0 (34.0–66.0)	39.0 (30.0–52.3)	T1–T2	− 0.74	** <.001**
				T1–T3	− 0.70	** <.001**
				T2–T3	− 0.44	** <.001**
Drowsiness	15.0 (11.0–19.0)	11.0 (9.0–17.0)	10.0 (7.0–14.3)	T1–T2	− 0.60	** <.001**
				T1–T3	− 0.62	** <.001**
				T2–T3	− 0.36	**.015**
Instability	8.0 (5.8–11.0)	6.0 (5.0–10.0)	6.0 (5.0–8.0)	T1–T2	− 0.56	** <.001**
				T1–T3	− 0.48	** <.001**
				T2–T3	− 0.23	.207
Uneasiness	9.0 (7.0–13.0)	8.0 (6.0–11.0)	7.0 (6.0–10.0)	T1–T2	− 0.54	** <.001**
				T1–T3	− 0.45	** <.001**
				T2–T3	− 0.17	.531
Local pain or dullness	11.0 (8.0–17.0)	8.0 (6.0–14.5)	7.0 (6.0–11.0)	T1–T2	− 0.71	** <.001**
				T1–T3	− 0.77	** <.001**
				T2–T3	− 0.51	** <.001**
Eyestrain	13.0 (8.8–18.0)	10.0 (6.8–15.0)	6.5 (5.0–11.0)	T1–T2	− 0.53	** <.001**
				T1–T3	− 0.69	** <.001**
				T2–T3	− 0.56	** <.001**

Notes: Continuous variables were presented as medians (interquartile range). Statistically significant values were marked in bold

Table 3 shows the results for fatigue recovery in the mixed model used to test Hypothesis 2. There were no significant differences in the total scores between the conditions. However, there was a significant main effect on the “local pain or dullness” sub-factor, for which the intervention condition showed significantly higher fatigue recovery than the control condition (*F*_[1, 61]_ = 5.46, partial *η*^*2*^ = 0.08, *P* = .023). Thus, Hypothesis 2 was partially supported. No significant interactions between condition and age were observed, which demonstrates that the effect of intervention on fatigue recovery had a consistent trend across all age groups (Additional file 4).

**Table 3:** Mixed effects model: fatigue recovery

**Changes in each fatigue score**	Model 1 (condition only)			Model 2 (adjusted family and night-shift napping)		
	*F* (df)	Partial *η*^*2*^	*P*	*F* (df)	Partial *η*^*2*^	*P*
**Main effect: condition**						
Total score	0.16 (1, 61)	0.00	.686	0.91 (1, 65)	0.01	.345
Drowsiness	0.14 (1, 61)	0.00	.710	0.09 (1, 65)	0.00	.759
Instability	0.38 (1, 60)	0.01	.538	0.56 (1, 66)	0.01	.456
Uneasiness	0.83 (1, 58)	0.01	.367	0.16 (1, 64)	0.00	.694
Local pain or dullness	5.46 (1, 61)	0.08	**.023**	6.29 (1, 68)	0.08	**.015**
Eyestrain	0.04 (1, 60)	0.00	.846	0.20 (1, 64)	0.00	.658
**Main effect: family**						
Total score	-	-	-	2.86 (1, 95)	0.03	.094
Drowsiness	-	-	-	0.79 (1, 95)	0.01	.375
Instability	-	-	-	0.00 (1, 87)	0.00	.987
Uneasiness	-	-	-	2.60 (1, 88)	0.03	.110
Local pain or dullness	-	-	-	3.62 (1, 92)	0.04	.060
Eyestrain	-	-	-	4.49 (1, 92)	0.05	**.037**
**Main effect: night-shift napping**						
Total score	-	-	-	3.75 (1, 115)	0.03	.055
Drowsiness	-	-	-	1.75 (1, 115)	0.01	.188
Instability	-	-	-	0.48 (1, 108)	0.00	.488
Uneasiness	-	-	-	4.76 (1, 111)	0.04	**.031**
Local pain or dullness	-	-	-	3.36 (1, 112)	0.03	.069
Eyestrain	-	-	-	3.83 (1, 113)	0.03	.053
**Interaction: condition × family**						
Total score	-	-	-	1.52 (1, 65)	0.02	.222
Drowsiness	-	-	-	0.33 (1, 65)	0.01	.565
Instability	-	-	-	2.17 (1, 65)	0.03	.146
Uneasiness	-	-	-	0.00 (1, 64)	0.00	.958
Local pain or dullness	-	-	-	5.55 (1, 68)	0.08	**.021**
Eyestrain	-	-	-	4.13 (1, 64)	0.06	**.046**
**Interaction: condition × night-shift napping**						
Total score	-	-	-	0.09 (1, 67)	0.00	.770
Drowsiness	-	-	-	0.00 (1, 67)	0.00	.997
Instability	-	-	-	0.84 (1, 69)	0.01	.363
Uneasiness	-	-	-	0.11 (1, 66)	0.00	.736
Local pain or dullness	-	-	-	0.50 (1, 70)	0.01	.480
Eyestrain	-	-	-	0.02 (1, 66)	0.00	.882
**Interaction: family × night-shift napping**						
Total score	-	-	-	1.53 (1, 115)	0.01	.219
Drowsiness	-	-	-	0.21 (1, 115)	0.00	.648
Instability	-	-	-	0.58 (1, 108)	0.01	.449
Uneasiness	-	-	-	1.47 (1, 110)	0.01	.229
Local pain or dullness	-	-	-	4.03 (1, 112)	0.03	**.047**
Eyestrain	-	-	-	3.16 (1, 113)	0.03	.078
**Interaction: condition × family × night-shift napping**						
Total score	-	-	-	0.16 (1, 66)	0.00	.694
Drowsiness	-	-	-	1.21 (1, 66)	0.02	.275
Instability	-	-	-	0.74 (1, 69)	0.01	.392
Uneasiness	-	-	-	0.44 (1, 66)	0.01	.509
Local pain or dullness	-	-	-	1.08 (1, 70)	0.02	.303
Eyestrain	-	-	-	3.99 (1, 66)	0.06	**.049**

Notes: “Changes in each fatigue score” represents the difference between the fatigue score immediately after the night shift and that on getting up from nighttime sleep on the day after the night shift, thus representing fatigue recovery. “Family” refers to being married or having dependent children, implying family and domestic responsibilities at home. Statistically significant values were marked in bold

### First and main sleep episodes

Of the 62 nurses, 56 (90.3%) obtained a first sleep episode in the control condition (no sleep = 6, missing = 0) and 49 (79.0%) in the intervention condition (no sleep = 8, missing = 5). A total of 61 nurses (98.4%) obtained the main sleep episode in the control condition (no sleep = 0, missing = 1) and 58 (93.5%) in the intervention condition (no sleep = 0, missing = 4). Excluding cases with missing data, all participants who had a first sleep episode subsequently experienced a distinct main sleep episode.

Examining Hypothesis 3, the intervention condition significantly delayed the start (*r* = 0.42, *P* = .005) and end (*r* = 0.34, *P* = .021) of TIB in the first sleep episode compared to the control condition (Table 4). However, comparisons of the main sleep episodes between the two conditions revealed no differences in any of the sleep parameters.

**Table 4:** Comparisons of first and main sleep episodes between the two conditions

	Control (*n* = 62)	Intervention (*n* = 62)	*r*	*P*
**First sleep episodes**	*n* = 56 ^a^	*n* = 49 ^b^		
Start of TIB [hh:mm]	12:05 (11:13–13:55)	13:17 (12:22–14:29)	0.42	**.005**
TIB [min]	261.0 (157.5–328.0)	222.0 (164.0–330.0)	0.02	.889
SL [min]	12.0 (4.0–29.5)	14.0 (6.0–26.0)	0.01	.953
TST [min]	196.0 (110.5–265.0)	168.0 (116.0–245.0)	0.03	.835
SE [%]	75.3 (66.4–82.7)	73.1 (61.5–84.8)	0.10	.509
WASO [min]	26.0 (6.0–56.0)	24.0 (14.0–64.0)	0.19	.201
BOT [min]	6.0 (4.0–10.0)	6.0 (4.0–10.0)	0.02	.875
End of TIB [hh:mm]	16:23 (15:20–18:38)	17:39 (16:10–19:02)	0.34	**.021**
**Main sleep episodes**	*n* = 61 ^c^	*n* = 58 ^d^		
Start of TIB [hh:mm]	24:04 (22:44–25:16)	23:39 (22:16–24:40)	0.12	.357
TIB [min]	536.0 (440.0–640.0)	553.0 (466.0–644.5)	0.15	.266
SL [min]	12.0 (4.0–29.0)	14.0 (6.0–36.0)	0.04	.750
TST [min]	404.0 (336.0–475.0)	405.0 (340.5–492.5)	0.15	.248
SE [%]	75.1 (65.6–82.1)	75.3 (66.4–85.5)	0.13	.324
WASO [min]	94.0 (62.0–150.0)	101.0 (45.0–151.0)	0.01	.971
BOT [min]	6.0 (4.0–12.0)	6.0 (6.0–10.5)	0.04	.764
End of TIB [hh:mm]	9:04 (7:24–10:25)	8:37 (7:33–10:17)	0.17	.207

Notes: Continuous variables were presented as medians (interquartile ranges). Statistically significant values were marked in bold

Abbreviations: *TIB*, the total time a person spent lying in bed; *SL*, the time from lying to falling asleep; *TST*, the sum of periods a person was asleep between the start and end of TIB; *SE*, the ratio of TST to TIB; *WASO*, the sum of time spent awake during a sleep; *BOT*, the time between waking up and getting up

^a^No sleep = 6, missing data = 0

^b^No sleep = 8, missing data = 5

^c^No sleep = 0, missing data = 1

^d^No sleep = 0, missing data = 4

### Change of daily activities and sleep episodes after taking a nap or break

**Fig. 4:**
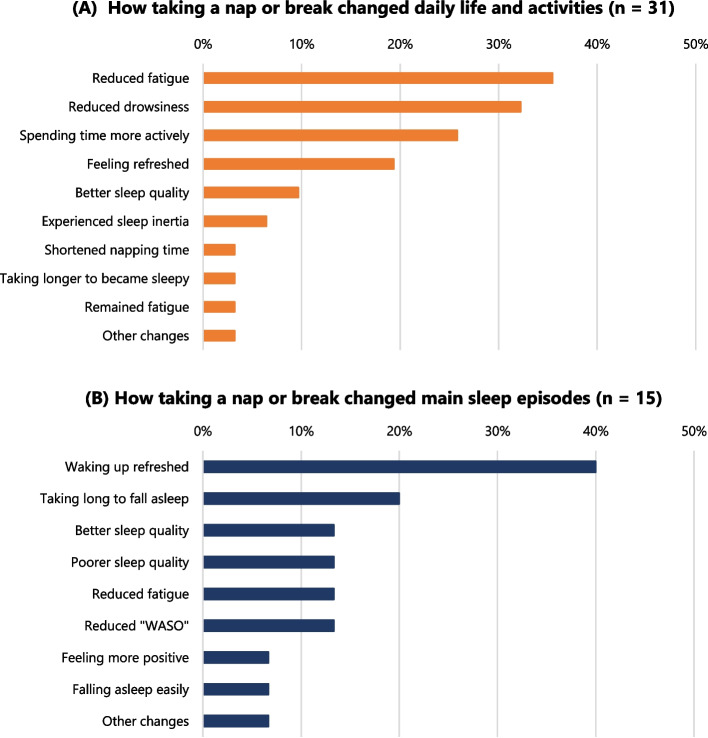
Change of daily activities and sleep episodes after taking a nap or break

Exactly half the nurses (*n* = 31) reported that taking a nap or break changed their daily lives and activities after the night shift. The percentages of responses for each category are presented in Fig. 4A. In addition, 24.2% (*n* = 15) of nurses reported that their main sleep episodes were affected by taking a nap or break. These percentages are illustrated in Fig. 4B.

### Exploratory subgroup analysis

**Fig. 5:**
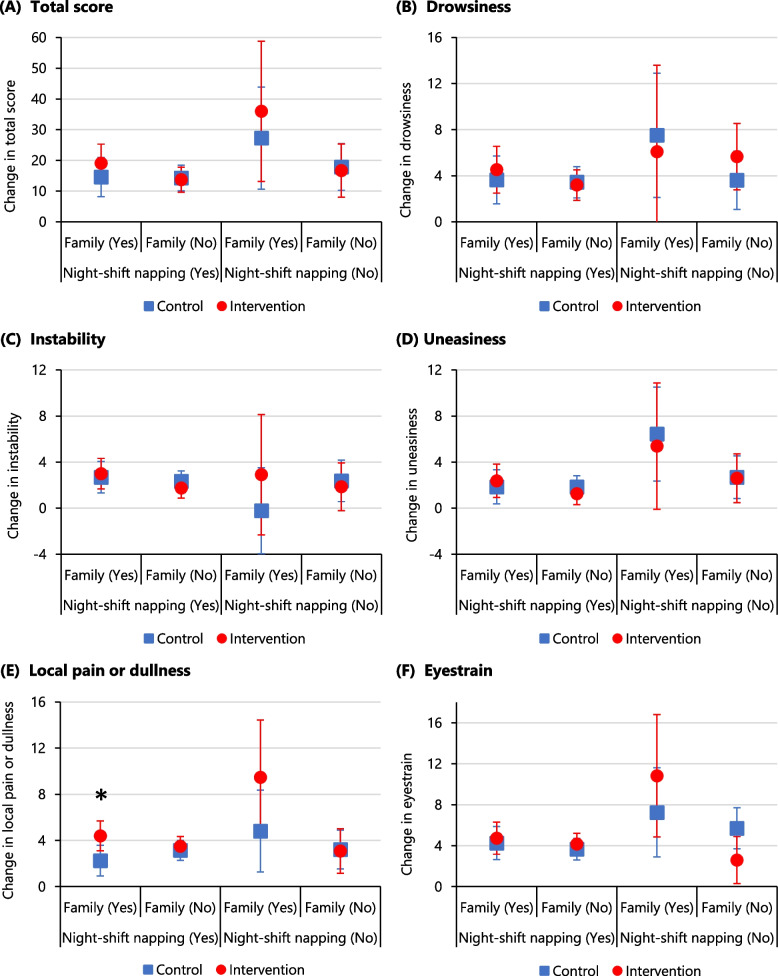
Adjusted recovery of fatigue by intervention condition, family, and night-shift napping Notes: Circles and squares represent the least squares means estimated by the mixed model. Changes in fatigue (vertical axis) are interpreted such that higher scores reflect higher levels of recovery from fatigue. Error bars represent 95% confidence interval. “Family” refers to being married or having dependent children, implying family and domestic responsibilities at home ^*^P = .007 (Bonferroni correction)

In the mixed model that considered the three factors of condition, family responsibilities, and night-shift napping, there were no significant main effects or interactions for the total scores (Table 3, Fig. 5A). However, there was a significant main effect of condition (*F*_[1, 68]_ = 6.29, partial* η*^*2*^ = 0.08, *P* = .015) and an interaction between condition and family responsibilities (*F*_[1, 68]_ = 5.55, partial* η*^*2*^ = 0.08, *P* = .021) for the “local pain or dullness” sub-factor (Fig. 5E). Multiple comparisons revealed that nurses who took naps during the night shift and had family responsibilities had higher fatigue recovery in the intervention condition than in the control condition (*P* = .007; Fig. 5E). There was also a significant main effect of family responsibilities on “eyestrain” (*F*_[1, 92]_ = 4.49, partial* η*^*2*^ = 0.05, *P* = .037), an interaction between condition and family responsibilities (*F*_[1, 64]_ = 4.13, partial* η*^*2*^ = 0.06, *P* = .046), and an interaction of condition, family responsibilities, and napping (*F*_[1, 66]_ = 3.99, partial* η*^*2*^ = 0.06, *P* = .049; Fig. 5F). Multiple comparisons showed no significant differences between any levels.

## Discussion

This study investigated the effects of taking a nap or break immediately after the night shift on nurses’ fatigue and sleep compared to going home as usual. All nurses in the intervention group took a break, and 79.0% also took a nap. Taking a nap or break significantly reduced fatigue levels immediately after the night shift. Although no significant difference in recovery of total fatigue scores was observed between the two conditions, recovery of “local pain or dullness” was significantly higher in the intervention condition. Furthermore, the intervention condition significantly delayed the timing of the first sleep episode compared to the control condition, whereas no significant differences were observed in the main sleep parameters. Among nurses who reported changes in their daily lives, activities, or sleep due to the intervention, approximately 20–40% described positive effects such as “reduced fatigue” and “spending time more actively.” However, approximately 5–10% reported negative effects. These findings suggest that nap or break immediately after night shift may help reduce fatigue and influence activity levels, although further research is needed to clarify which napping conditions are most beneficial and for whom.

Taking a nap after the night shift was expected to reduce fatigue before returning home [16], and our results supported this hypothesis. All fatigue scores after taking a nap or break were significantly lower than those immediately after the night shift, with large effect sizes. We also observed that the total fatigue score (median [IQR], 43.0 [34.0–66.0]) was lower in the intervention condition than in the control condition (50.0 [39.5–79.3]) before returning home (Additional file 5). Laboratory studies have demonstrated that prolonged wakefulness (e.g., 17–19 h) yields cognitive deficits comparable to a blood alcohol concentration of 0.05% [38, 39]; hence, nurses have a high risk of traffic accidents after long night shifts [14, 15]. Our findings suggest a means of preventing traffic accidents among shift nurses.

The results partially supported Hypothesis 2, in that taking a nap or break immediately after the night shift enhanced post-night shift recovery from “local pain or dullness,” one of the symptoms of work-related fatigue. We suggest that this result may be explained by the additional time available for activity with lower fatigue levels after the night shift. Figure 4A showed that some nurses felt that taking a nap or break “reduced fatigue” and “reduced drowsiness” during their non-working hours. These results suggest a way of preventing maladaptive chronic fatigue and fatigue/sleep-related traffic accidents. Moreover, some nurses were able to “spend time more actively” or “felt refreshed,” contributing to their well-being after the night shift [28]. Spending time more actively during free time is significantly associated with lower maladaptive chronic fatigue symptoms [22]. Hence, taking a nap or break immediately after the night shift appears to positively affect nurses’ recovery from fatigue according to their subjective assessments.

Furthermore, we conducted subgroup analyses to examine changes in outcomes due to family responsibilities [21], a factor impeding recovery from fatigue, and napping during the night shift [12], which facilitates recovery from fatigue. The findings demonstrated a significant main effect of condition and an interaction between condition and family responsibilities for local pain or dullness, with medium effect sizes. Thus, taking a nap immediately after the night shift may be particularly effective for nurses with family responsibilities. Marital status is associated with increased fatigue among nurses working in a two-shift system [7]. Specifically, nurses with lower intershift recovery included a higher proportion of married nurses than those with higher intershift recovery [40]. These studies suggest that domestic obligations caused by having a spouse and children can restrict intershift recovery [21]. Therefore, we inferred that taking a nap or break was practical for nurses with family responsibilities as it allowed them to recover during non-working hours without being affected by home demands.

Moreover, an interaction was identified between condition, family, and night-shift napping with respect to recovery from the “eyestrain” sub-factor. For nurses who could nap during the night shift, which facilitated recovery from fatigue [12], the recovery between conditions was similar regardless of family responsibilities. However, among nurses who could not nap, those with family responsibilities tended to benefit more from taking a nap or break. In future, if we can verify the differences in the effects of fatigue recovery caused by family responsibilities and night-shift napping, our intervention could be targeted at these individuals.

### Strengths and limitations

To the best of our knowledge, this is the first study to verify the effect of napping immediately after the night shift in a controlled napping environment. Furthermore, the study was performed with nurses in a real setting rather than a laboratory environment, to ensure the ecological validity of the results.

This study has several limitations. First, no measurement point corresponding to the post-nap or break measurement in the intervention condition was included in the control condition (i.e., 30-min after the night shift). We assumed that having nurses wait for 30-min immediately after the night shift would increase fatigue and drowsiness and could harm their health. Therefore, the present design was based on ethical considerations. However, the lack of a time-matched assessment in the control group makes isolation of the intervention effect from natural recovery over time difficult, and the precise effect size cannot be determined. Second, the small sample size and possible between-group imbalances precluded detailed examination of the differences between nurses napped and those who only took a break under the intervention condition. Third, our results have limited generalizability to nurses working shorter night shifts (e.g., 8-h and 12-h), in which fatigue levels are assumed to differ. Fourth, although we explored possible heterogeneity related to family responsibilities and napping during night shifts, we were unable to examine other potential confounding factors due to the limited sample size. Specifically, factors such as the timing, quality, and quantity of naps during and after night shifts are likely to influence the outcomes. Therefore, further studies with larger sample sizes are required to validate the results for other shift patterns while controlling for various confounding factors. Finally, the long-term effect of taking a nap or break after a night shift remains unknown. Since the intervention in this study involved only one night shift, future research should examine the effects of repeated intervention on longer-term outcomes such as chronic fatigue.

## Conclusions

This study examined the effects of taking a nap or break immediately after a long night shift on nurses’ fatigue recovery and sleep episodes. The results indicated that taking a nap or break immediately after the night shift significantly reduced fatigue before returning home. The degree of recovery from “local pain or dullness,” which is one of the symptoms of work-related fatigue, was significantly higher than that among nurses who went home as usual. Furthermore, this intervention delayed the timing of the first sleep episode compared to the control condition; however, no significant differences were observed in the main sleep parameters. These findings suggest that a nap or break immediately after a night shift may help reduce fatigue and influence activity levels. Therefore, implementing a nap or break following the night shift could serve as an effective countermeasure to address fatigue/sleep-related issues among nurses. This approach could be recognized as a valuable component of comprehensive fatigue risk management by both nurses and managers.

## Implications

Solutions to address shift nurses’ fatigue, sleep deprivation, and related problems are needed in healthcare and society. Existing interventions include napping during night shifts, modifying shift schedules and patterns, light exposure, exercise, aromatherapy, and melatonin [2, 29]. However, the level of evidence remains low to moderate, and further empirical studies are required [2, 29]. This study suggested that taking a nap or break immediately after the night shift may help nurses recover from fatigue and sleep deprivation. Our findings may be useful for nurses who feel highly fatigued or drowsy after the night shift, who drive their cars after the night shift, or who find it challenging to recover at home. Nursing managers should recognize taking a nap or break immediately after the night shift as a viable option in comprehensive fatigue risk management [2]. Thus, we suggest that it is essential to develop napping environments [24, 25] that can provide a comfortable nap or break in clinical settings.

## Supplementary Information

Additional file 1. TREND statement checklist

Additional file 2. Comparison of two conditions: Time course of fatigue during the night shift

Additional file 3. Comparison of nurses who napped vs. those who did not nap in the nap environment control system

Additional file 4. Exploratory analysis of the effect of the interactions between condition and age on fatigue recovery

Additional file 5. Descriptive statistics: Time course of post-night shift fatigue in the two conditions

## Data Availability

The data that support the findings of this study are available on request from the corresponding author. The data are not publicly available due to privacy or ethical restrictions.

## Abbreviations

ANCOVA: Analysis of covariance
BOT: Bed-out time
IQR: Interquartile ranges
SE: Sleep efficiency
SL: Sleep latency
SSA: SleepSign Act
TIB: Time in bed
TST: Total sleep time
WASO: Wake after sleep onset
