# Supplementary material for: Effects of taking a nap or break immediately after night shift on nurses’ fatigue recovery and sleep episodes: a quasi-experimental study
Source: J Physiol Anthropol. 2025 Jul 15;44:21. doi: 10.1186/s40101-025-00399-2 (PMC12261775; doi:10.1186/s40101-025-00399-2)
Supplement: Supplementary file 2 — Additional file 2. Comparison of two conditions: Time course of fatigue during the night shift [file 40101_2025_399_MOESM2_ESM.docx]

**Additional file 2.** Comparison of two conditions: Time course of fatigue during the night shift

|  | Time | | | | Main effect | | | | Interaction | |
| --- | --- | --- | --- | --- | --- | --- | --- | --- | --- | --- |
|  |  |  |  |  | Condition | | Time | | Condition × Time | |
|  | Beginning of the night shift | Before nap/break during the night shift | After nap/break during the night shift | Immediately after the night shift | *F* (df) | *P* | *F* (df) | *P* | *F* (df) | *P* |
| **Total score** |  |  |  |  |  |  |  |  |  |  |
| Intervention | 47.1 (41.7–52.4) | 56.3 (50.9–61.7) | 59.1 (53.8–64.5) | 59.5 (54.2–64.9) | 0.03  (1, 427) | .847 | 57.83  (3, 427) | <.001 | 0.18  (3, 427) | .907 |
| Control | 46.2 (40.9–51.6) | 56.5 (51.1–61.8) | 59.8 (54.4–65.1) | 59.0 (53.6–64.3) |  |  |  |  |  |  |
| **Drowsiness** |  |  |  |  |  |  |  |  |  |  |
| Intervention | 10.0 (8.7–11.3) | 13.0 (11.7–14.3) | 15.0 (13.7–16.3) | 15.0 (13.7–16.3) | 0.02  (1, 427) | .891 | 70.96  (3, 427) | <.001 | 1.47  (3, 427) | .221 |
| Control | 10.1 (8.8–11.5) | 13.8 (12.5–15.2) | 14.9 (13.6–16.2) | 14.2 (12.9–15.6) |  |  |  |  |  |  |
| **Instability** |  |  |  |  |  |  |  |  |  |  |
| Intervention | 10.0 (8.8–11.2) | 9.9 (8.7–11.1) | 10.0 (8.8–11.2) | 9.4 (8.2–10.6) | 0.75  (1, 427) | .387 | 1.50  (3, 427) | .213 | 0.31  (3, 427) | .816 |
| Control | 9.6 (8.4–10.8) | 9.6 (8.4–10.8) | 10.0 (8.8–11.2) | 9.4 (8.2–10.6) |  |  |  |  |  |  |
| **Uneasiness** |  |  |  |  |  |  |  |  |  |  |
| Intervention | 8.7 (7.7–9.8) | 9.8 (8.7–10.8) | 10.5 (9.5–11.6) | 10.1 (9.1–11.2) | 0.08  (1, 427) | .771 | 23.38  (3, 427) | <.001 | 0.85  (3, 427) | .465 |
| Control | 8.4 (7.3–9.4) | 9.7 (8.6–10.7) | 10.9 (9.9–12.0) | 10.5 (9.4–11.5) |  |  |  |  |  |  |
| **Local pain or dullness** |  |  |  |  |  |  |  |  |  |  |
| Intervention | 9.4 (8.1–10.6) | 11.8 (10.6–13.0) | 11.3 (10.1–12.5) | 12.4 (11.2–13.6) | 0.18  (1, 427) | .672 | 57.64  (3, 427) | <.001 | 0.37  (3, 427) | .776 |
| Control | 9.3 (8.1–10.5) | 11.4 (10.2–12.6) | 11.4 (10.2–12.6) | 12.4 (11.2–13.6) |  |  |  |  |  |  |
| **Eyestrain** |  |  |  |  |  |  |  |  |  |  |
| Intervention | 9.0 (7.7–10.2) | 11.8 (10.6–13.1) | 12.3 (11.1–13.6) | 12.6 (11.4–13.9) | 0.00  (1, 427) | .999 | 56.67  (3, 427) | <.001 | 0.19  (3, 427) | .904 |
| Control | 8.8 (7.5–10.0) | 12.0 (10.7–13.2) | 12.5 (11.3–13.8) | 12.5 (11.2–13.7) |  |  |  |  |  |  |

***Notes***: Continuous variables were presented as least squares means (95% confidence intervals) by the linear mixed model.
