# Supplementary material for: Effects of taking a nap or break immediately after night shift on nurses’ fatigue recovery and sleep episodes: a quasi-experimental study
Source: J Physiol Anthropol. 2025 Jul 15;44:21. doi: 10.1186/s40101-025-00399-2 (PMC12261775; doi:10.1186/s40101-025-00399-2)
Supplement: Supplementary file 3 — Additional file 3. Comparison of nurses who napped vs. those who did not nap in the nap environment control system [file 40101_2025_399_MOESM3_ESM.docx]

**Additional file 3.** Comparison of nurses who napped vs. those who did not nap in the nap environment control system

|  | Nap (n = 49) | No nap (break only: n = 13) | *P* ^a^ |
| --- | --- | --- | --- |
| Age (years) | 34.4 (27.5–42.0) | 34.0 (29.0–41.5) | .808 |
| Years as nurse (years) | 9.9 (4.8–18.2) | 9.6 (5.7–10.8) | .568 |
| BMI (kg/m^2^) | 21.2 (19.3–22.1) | 21.0 (18.8–25.1) | .999 |
| Sex (female) | 47 (95.9%) | 13 (100%) | .459 |
| Family and domestic responsibilities at home (yes) | 15 (30.6%) | 3 (23.1%) | .595 |
| Married (yes) | 14 (28.6%) | 2 (15.4%) | .334 |
| Child rearing (yes) | 11 (22.4%) | 2 (15.4%) | .578 |
| Cumulative steps during night shift (steps) | 9549.0 (7929.5–11746.8) | 9402.0 (8508.0–10721.5) | .909 |
| Resting time during night shift (min) | 120.0 (120.0–157.5) | 120.0 (100.0–155.0) | .446 |
| Napping during night shift (yes) | 43 (87.8%) | 12 (92.3%) | .645 |
| Start of TIB (hh:mm) | 26:02 (24:30–27:10) | 27:07 (25:47–27:34) | .142 |
| TIB (min) | 118.0 (100.0–154.5) | 103.0 (74.5–130.5) | .116 |
| SL (min) | 18.0 (6.0–30.0) | 15.0 (7.0–19.5) | .226 |
| TST (min) | 80.0 (62.0–104.5) | 74.0 (49.5–101.0) | .423 |
| SE (%) | 68.8 (57.2–80.1) | 69.9 (58.3–90.0) | .795 |
| WASO (min) | 6.0 (0.0–14.0) | 8.0 (0.0–25.0) | .606 |
| BOT (min) | 4.0 (4.0–6.5) | 4.0 (4.0–6.0) | .920 |
| End of TIB (hh:mm) | 28:51 (26:26–29:22) | 28:48 (27:04–29:30) | .771 |
| First sleep episodes |  |  |  |
| Start of TIB [hh:mm] | 13:14 (12:14–14:04) | 13:42 (12:37–15:27) | .353 |
| TIB [min] | 249.0 (190.5–341.5) | 190.0 (130.0–284.0) | .063 |
| SL [min] | 17.0 (6.5–33.5) | 10.0 (5.0–20.0) | .069 |
| TST [min] | 182.0 (121.0–270.0) | 118.0 (103.0–223.0) | .141 |
| SE [%] | 73.8 (58.4–85.6) | 72.7 (63.8–82.3) | .777 |
| WASO [min] | 20.0 (14.0–71.0) | 26.0 (12.0–44.0) | .626 |
| BOT [min] | 4.0 (4.0–8.5) | 6.0 (4.0–7.0) | .660 |
| End of TIB [hh:mm] | 17:44 (16:13–19:02) | 17:34 (16:09–19:03) | .903 |
| Main sleep episodes |  |  |  |
| Start of TIB [hh:mm] | 23:30 (21:57–24:42) | 23:49 (22:44–24:20) | .666 |
| TIB [min] | 553.0 (440.5–646.5) | 550.0 (497.5–603.0) | .855 |
| SL [min] | 14.0 (5.5–36.0) | 15.0 (7.0–46.5) | .700 |
| TST [min] | 394.0 (340.5–492.5) | 430.0 (336.0–520.5) | .751 |
| SE [%] | 73.7 (66.4–85.0) | 81.9 (60.4–85.7) | .666 |
| WASO [min] | 104.0 (42.0–143.5) | 83.0 (50.0–169.0) | .924 |
| BOT [min] | 6.0 (6.0–12.0) | 7.0 (6.0–10.0) | .539 |
| End of TIB [hh:mm] | 8:29 (7:27–10:05) | 9:08 (8:02–10:51) | .382 |
| Fatigue recovery (after night shift−after using nap environment control system) |  |  |  |
| Total score | 9.0 (2.5–13.0) | 10.0 (2.5–20.5) | .383 |
| Drowsiness | 2.0 (0.0–5.0) | 2.0 (0.0–5.0) | .551 |
| Instability | 1.0 (0.0–3.0) | 1.0 (0.0–2.5) | .246 |
| Uneasiness | 1.0 (0.0–2.0) | 1.0 (0.5–4.0) | .206 |
| Local pain or dullness | 2.0 (0.0–3.0) | 2.0 (0.0–3.0) | .592 |
| Eyestrain | 1.0 (0.0–3.5) | 1.0 (0.0–5.0) | .340 |
| Fatigue recovery (after night shift−getting up the day after night shift) |  |  |  |
| Total score | 15.0 (3.5–30.0) | 15.0 (−0.5–32.0) | .709 |
| Drowsiness | 3.0 (1.0–9.0) | 3.0 (−1.0–9.0) | .748 |
| Instability | 1.0 (0.0–4.0) | 1.0 (0.0–4.5) | .393 |
| Uneasiness | 1.0 (0.0–3.0) | 3.0 (0.0–4.5) | .368 |
| Local pain or dullness | 3.0 (1.0–6.0) | 2.0 (1.5–7.5) | .774 |
| Eyestrain | 3.0 (0.0–8.0) | 6.0 (1.0–11.0) | .596 |

***Notes***: Continuous variables were presented as medians (interquartile ranges), whereas categorical variables were described as N (%).

***Abbreviations***: TIB = the total time a person spent lying in bed; SL = the time from lying to falling asleep; TST = the sum of periods a person was asleep between the start and end of TIB; SE = the ratio of TST to TIB; WASO = the sum of time spent awake during a sleep; BOT = the time between waking up and getting up.

^a^ Mann–Whitney U test or Chi-squared test. Recovery from fatigue was analyzed using ANCOVA, adjusting for baseline scores.
