# Supplementary material for: Effects of taking a nap or break immediately after night shift on nurses’ fatigue recovery and sleep episodes: a quasi-experimental study
Source: J Physiol Anthropol. 2025 Jul 15;44:21. doi: 10.1186/s40101-025-00399-2 (PMC12261775; doi:10.1186/s40101-025-00399-2)
Supplement: Supplementary file 4 — Additional file 4. Exploratory analysis of the effect of the interactions between condition and age on fatigue recovery [file 40101_2025_399_MOESM4_ESM.docx]

**Additional file 4.** Exploratory analysis of the effect of the interactions between condition and age on fatigue recovery

| **Changes in each fatigue scores** | *F* (df) | Partial *η^2^* | *P* |
| --- | --- | --- | --- |
| **Main effect: condition** |  |  |  |
| Total score | 0.15 (1, 59) | 0.00 | .696 |
| Drowsiness | 0.54 (1, 59) | 0.01 | .467 |
| Instability | 0.39 (1, 58) | 0.01 | .533 |
| Uneasiness | 1.16 (1, 57) | 0.02 | .285 |
| Local pain or dullness | 5.04 (1, 59) | 0.08 | **.029** |
| Eyestrain | 0.03 (1, 58) | 0.00 | .857 |
| **Main effect: age** |  |  |  |
| Total score | 2.60 (2, 59) | 0.08 | .083 |
| Drowsiness | 0.32 (2, 58) | 0.01 | .731 |
| Instability | 3.08 (2, 58) | 0.10 | .054 |
| Uneasiness | 1.98 (2, 57) | 0.06 | .148 |
| Local pain or dullness | 6.76 (2, 59) | 0.19 | **.002** |
| Eyestrain | 1.08 (2, 58) | 0.04 | .347 |
| **Interaction: condition × age** |  |  |  |
| Total score | 2.13 (2, 59) | 0.07 | .128 |
| Drowsiness | 1.72 (2, 58) | 0.06 | .189 |
| Instability | 3.00 (2, 58) | 0.09 | .058 |
| Uneasiness | 0.47 (2, 57) | 0.02 | .627 |
| Local pain or dullness | 0.79 (2, 59) | 0.03 | .458 |
| Eyestrain | 2.14 (2, 58) | 0.07 | .127 |

***Notes***: A mixed model was employed to compare the degree of fatigue recovery between the two conditions, adjusting for age and baseline scores.

Statistically significant values were marked in bold.
