# Supplementary material for: Effects of taking a nap or break immediately after night shift on nurses’ fatigue recovery and sleep episodes: a quasi-experimental study
Source: J Physiol Anthropol. 2025 Jul 15;44:21. doi: 10.1186/s40101-025-00399-2 (PMC12261775; doi:10.1186/s40101-025-00399-2)
Supplement: Supplementary file 5 — Additional file 5. Descriptive statistics: Time course of post-night shift fatigue in the two conditions [file 40101_2025_399_MOESM5_ESM.docx]

**Additional file 5.** Descriptive statistics: Time course of post-night shift fatigue in the two conditions

|  | Immediately after the night shift | After taking a 30-minute nap/break | Getting up from nighttime sleep on the day after the night shift |
| --- | --- | --- | --- |
| **Total score** |  |  |  |
| Intervention | 55.0 (42.0–80.0) | 43.0 (34.0–66.0) | 39.0 (30.0–52.3) |
| Control | 50.0 (39.5–79.3) |  | 40.0 (31.0–51.5) |
| **Drowsiness** |  |  |  |
| Intervention | 15.0 (11.0–19.0) | 11.0 (9.0–17.0) | 10.0 (7.0–14.3) |
| Control | 13.5 (9.0–19.3) |  | 11.0 (7.0–14.0) |
| **Instability** |  |  |  |
| Intervention | 8.0 (5.8–11.0) | 6.0 (5.0–10.0) | 6.0 (5.0–8.0) |
| Control | 7.0 (6.0–11.3) |  | 6.0 (5.0–7.3) |
| **Uneasiness** |  |  |  |
| Intervention | 9.0 (7.0–13.0) | 8.0 (6.0–11.0) | 7.0 (6.0–10.0) |
| Control | 9.0 (6.0–13.0) |  | 7.0 (6.0–10.0) |
| **Local pain or dullness** |  |  |  |
| Intervention | 11.0 (8.0–17.0) | 8.0 (6.0–14.5) | 7.0 (6.0–11.0) |
| Control | 12.0 (7.0–17.0) |  | 8.0 (6.0–13.0) |
| **Eyestrain** |  |  |  |
| Intervention | 13.0 (8.8–18.0) | 10.0 (6.8–15.0) | 6.5 (5.0–11.0) |
| Control | 11.0 (8.5–17.3) |  | 7.0 (5.0–11.0) |

***Notes***: Continuous variables were presented as medians (interquartile ranges).

Wilcoxon signed-rank test showed no significant differences between two conditions for all fatigue scores at immediately after the night shift and getting up from nighttime sleep on the day after the night shift.
